# Neutrophil-derived miR-223 as local biomarker of bacterial peritonitis

**DOI:** 10.1038/s41598-019-46585-y

**Published:** 2019-07-12

**Authors:** Amy C. Brook, Robert H. Jenkins, Aled Clayton, Ann Kift-Morgan, Anne-Catherine Raby, Alex P. Shephard, Barbara Mariotti, Simone M. Cuff, Flavia Bazzoni, Timothy Bowen, Donald J. Fraser, Matthias Eberl

**Affiliations:** 1 Division of Infection and Immunity, School of Medicine, Cardiff University, Cardiff, United Kingdom; 2 Wales Kidney Research Unit, Heath Park Campus, Cardiff, United Kingdom; 3 Division of Cancer and Genetics, School of Medicine, Cardiff University, Cardiff, United Kingdom; 4 Department of Medicine, Section of General Pathology, University of Verona, Verona, Italy; 5 Directorate of Nephrology and Transplantation, Cardiff and Vale University Health Board, University Hospital of Wales, Cardiff, United Kingdom; 6 Systems Immunity Research Institute, Cardiff University, Cardiff, United Kingdom

**Keywords:** Peritoneal dialysis, Infectious-disease diagnostics, Diagnostic markers

## Abstract

Infection remains a major cause of morbidity, mortality and technique failure in patients with end stage kidney failure who receive peritoneal dialysis (PD). Recent research suggests that the early inflammatory response at the site of infection carries diagnostically relevant information, suggesting that organ and pathogen-specific “immune fingerprints” may guide targeted treatment decisions and allow patient stratification and risk prediction at the point of care. Here, we recorded microRNA profiles in the PD effluent of patients presenting with symptoms of acute peritonitis and show that elevated peritoneal miR-223 and reduced miR-31 levels were useful predictors of bacterial infection. Cell culture experiments indicated that miR-223 was predominantly produced by infiltrating immune cells (neutrophils, monocytes), while miR-31 was mainly derived from the local tissue (mesothelial cells, fibroblasts). miR-223 was found to be functionally stabilised in PD effluent from peritonitis patients, with a proportion likely to be incorporated into neutrophil-derived exosomes. Our study demonstrates that microRNAs are useful biomarkers of bacterial infection in PD-related peritonitis and have the potential to contribute to disease-specific immune fingerprints. Exosome-encapsulated microRNAs may have a functional role in intercellular communication between immune cells responding to the infection and the local tissue, to help clear the infection, resolve the inflammation and restore homeostasis.

## Introduction

Peritoneal dialysis (PD) is an effective and life-saving renal replacement therapy for approximately 250,000 patients with end stage kidney failure worldwide^1^. Excess waste products are removed from the blood via natural ultrafiltration and osmosis across the semi-permeable peritoneal membrane into the dialysis solution instilled in the peritoneal cavity. However, despite the superior convenience and quality of life of PD compared to haemodialysis as alternative modality, infection and infection-related inflammation remain a major cause of morbidity and treatment failure in this patient group. The need to exchange the dialysis fluid, typically 3–4 times a day, requires the presence of a permanent catheter which greatly increases the risk of peritoneal infections, primarily by bacteria^1,2^. The accompanying inflammatory reaction can be detrimental for the patient, causing substantial acute morbidity and chronic scarring of the peritoneal membrane, and ultimately contributing to the deterioration of ultrafiltration and poor outcomes.

Current methods of identifying the causative pathogen through microbiological culture are slow and error-prone, and may lead to ambiguous or even negative culture results. In the absence of rapid and reliable diagnostic tests at the point of care, patients typically receive an empirical first-line treatment of broad-spectrum antibiotics, long before culture results become available and prescription can be tailored toward the causative organism^2^. This delay in giving the most appropriate treatment to patients, combined with the exposure to drugs that are not actually needed, adds to inflammation-related damage, avoidable drug-specific side effects, unnecessary health care costs and the ever-increasing emergence of multidrug resistant microbes^3^. However, despite the unmet need to provide early and targeted therapy, no major improvements in the diagnosis of PD-related peritonitis have been implemented in the clinic^1,4^. This is even more surprising given the landmark observation from 25 years ago that numbers of neutrophils and levels of CXCL8/IL-8 in the peritoneal effluent, biomarkers that are relatively easy to determine, are already elevated over baseline 1–2 days before presentation with full clinical symptoms of peritonitis^5,6^. These findings provide proof of concept that early diagnosis is possible, ideally even as home test for individuals feeling unwell but before developing a ‘cloudy’ effluent.

Our own recent research suggests that the inflammatory response evoked at the site of infection is sufficiently specific to be indicative of the type of causative pathogen, through a combination of pathogen-specific biomarkers (‘immune fingerprints’) triggered by certain microbes but not by others^3,7–9^. While most efforts have so far focused on the composition of the cellular infiltrate and soluble proteins, developing suitable high-affinity detection reagents for protein biomarkers can be challenging. In contrast, extracellular microRNAs are emerging as a new class of molecules with great potential as biomarkers in a range of pathologies. MicroRNAs are small, endogenous, non-coding, single-stranded RNA molecules that act as post-transcriptional regulators and are estimated to control the expression of more than half of all protein-coding genes in the human genome^10–12^. As they are involved in most cellular processes, local microRNAs are likely to play a role in the immune response to infection. Earlier work already showed that microRNAs are stable biomarkers in PD effluent and that molecules such as miR-21 and miR-31 are associated with inflammation-related fibrosis in the peritoneum^13^. We here undertook a comprehensive screening to identify microRNAs in PD effluent that are associated with episodes of bacterial peritonitis. Our findings shows that miR-223 in particular is a promising biomarker that is produced by neutrophils, increased in acute peritonitis, and associated with extracellular vesicles in infected PD effluent.

## Results

### Unbiased screening of infection-associated microRNAs in pooled patient samples

To study the role of microRNAs in PD-related peritonitis episodes, a TaqMan low density array (TLDA) was used to determine expression patterns of 377 microRNAs. As the majority of these microRNAs were below the detection limit in neat PD effluent (data not shown), a pre-amplification step was included that allowed successful detection of a total of 122 microRNAs. Effluent samples from three different groups of individuals receiving PD were investigated: uninfected stable patients who had not experienced an episode of peritonitis for at least 3 months prior to recruitment into this study, and those presenting with acute peritonitis that was subsequently confirmed by microbiological culture as infection by either coagulase-negative *Staphylococcus* (CNS) or *E*. *coli*. CNS and *E*. *coli* are the most common Gram-positive and Gram-negative organisms in PD patients, respectively, and cause the majority of peritonitis episodes at the local hospital; they were thus selected as well-defined representatives for the spectrum of Gram-positive and Gram-negative bacteria that may cause peritonitis in the PD community. All infected samples were from the first cloudy bag on day 1 of presentation with peritonitis, before antibiotic treatment commenced. Samples from ten patients were pooled for each group, to average out inter-sample variability^14^.

TLDA analysis revealed infection-dependent alterations in the abundance of nine peritoneal microRNAs, as defined by greater than two-fold changes in relative expression in cloudy effluent compared to stable samples, normalised to the global expression of all microRNAs (Table 1). Levels of miR-223, miR-139 and miR-197 were increased in both CNS infections (Gram^+^) and *E*. *coli* infections (Gram^−^). Levels of miR-27a were elevated in CNS but not in *E*. *coli* infections. Levels of miR-21 and miR-31 were decreased in both types of infections compared to uninfected samples; three further microRNAs (miR-199a, miR-100 and miR-99a) were especially low in *E*. *coli* infections. In preliminary experiments, detection of miR-139 and miR-197 in individual samples by RT-qPCR as a more accurate means of analysis did not confirm the infection-related increase observed in the TLDA screening (data not shown). For miR-99a, miR-100 and miR-199a, levels were generally low, at times even below the detection limit (data not shown). As consequence, miR-139, miR-197, miR-99a, miR-100 and miR-199a were all excluded from the subsequent analysis, and only miR-223, miR-21, miR-27a and miR-31 were taken forward for further validation.

**Table 1:** Identification of infection-associated microRNAs using Taqman Low Density Arrays.

microRNA	Gram^+^ infection	Gram^−^ infection
miR-223	+6.9	+13.1
miR-139-5p	+3.5	+8.3
miR-197	+2.2	+7.9
miR-27a	+4.8	+1.2
miR-21	−2.6	−1.4
miR-31	−1.9	−2.1
miR-199a-3p	−2.8	−12.3
miR-100	−3.7	−16.7
miR-99a	−1.3	−55.6

10 effluent samples each were pooled from stable non-infected individuals and from patients presenting with acute peritonitis that was subsequently confirmed as coagulase-negative *Staphylococcus* (Gram^+^) or *E*. *coli* infections (Gram^−^), and analysed using TLDA. microRNA levels in each group were normalised to the global expression levels across the plate, and are shown as relative values compared to levels in stable individuals as reference (set to 1.0). Only microRNAs that could be detected in all three groups were included in this analysis.

### Validation of TLDA results in individual patients

To confirm the expression patterns seen with pooled samples, miR-223, miR-21, miR-27a and miR-31 were subjected to a validation across a cohort of PD patients presenting at the University Hospital of Wales in Cardiff with peritonitis, between September 2008 and October 2018. Samples from 109 acutely ill PD patients were grouped according to the microbiological culture results received several days after presentation, into infections caused by Gram-positive organisms (*n* = 77), including CNS (*n* = 40), *Staphylococcus aureus* (*n* = 9) and *Streptococcus spp*. (*n* = 16); and those caused by Gram-negative organisms (*n* = 32), including *E*. *coli* (*n* = 18), other coliform species (*n* = 4) and *Pseudomonas spp*. (*n* = 3) (Supplemental Table S1). Effluent samples from 20 age and gender-matched stable PD patients served as controls.

**Figure 1:**
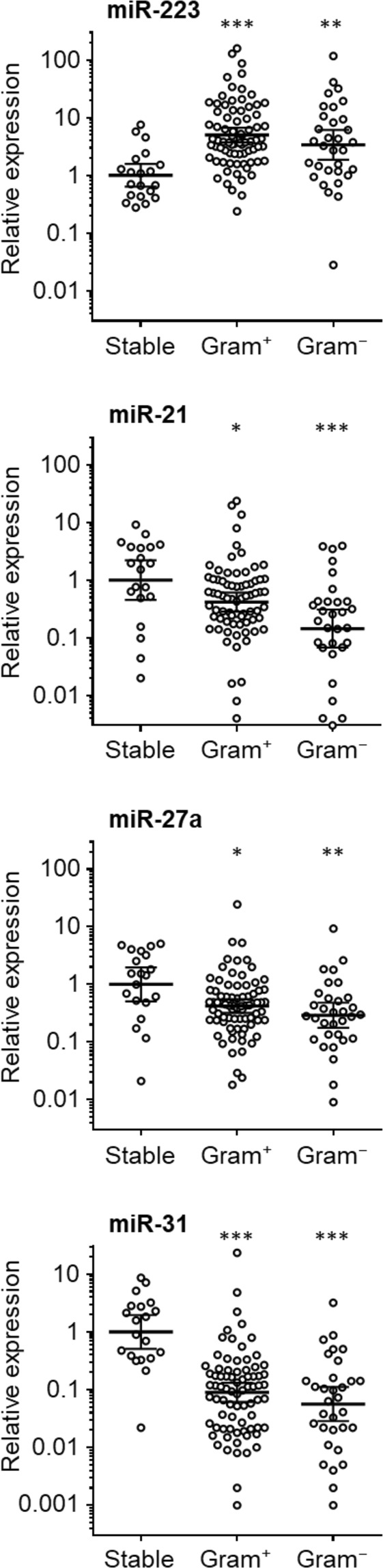
Peritoneal microRNAs in PD patients with and without acute bacterial peritonitis. Levels of miR-223, miR-21, miR-31 and miR-27a were measured in effluent samples from 20 stable PD patients, 76 patients with confirmed Gram^+^ infections and 31 with Gram^−^ infections, and normalised to snRNA U6. Each data point represents an individual patient; lines indicate geometric means and 95% confidence intervals. Data were analysed using Kruskal-Wallis tests combined with Dunn’s multiple comparisons tests versus stable controls.

As shown in Fig. 1, miR-223 levels were greatly increased during acute infection, compared to stable individuals, in accordance with the TLDA screening of pooled samples. Similarly, the miR-21 and miR-31 TLDA results could be validated, with lower levels of these microRNAs in both Gram^+^ and Gram^−^ peritonitis, compared to those without an infection. In contrast, the increase in miR-27a levels seen with pooled samples from CNS infections could not be replicated across the full patient cohort, instead miR-27a levels showed a significant decrease in both Gram^+^ and Gram^−^ infections (Fig. 1). Taken together, these findings broadly mirrored the trends from the TLDA screening data, with the exception of miR-27a, and established the diagnostic potential of extracellular microRNAs in patients presenting with acute PD-related peritonitis. Indeed, all four microRNAs reached areas under the curve (AUC) of >0.7 in a ROC analysis, with miR-223 and miR-31 being particularly good at distinguishing stable from infected samples and in combination reaching an AUC of 0.986 (Table 2; Supplemental Fig. S1). Power analyses of the ROC curve and precision-recall curves confirmed the validity of these AUC values (data not shown). Of note, miR-21 was the only microRNA found to discriminate between patients with confirmed Gram^+^ and Gram^−^ infections, albeit only with a very modest AUC of 0.636 (Supplemental Fig. S2).

**Table 2:** Peritoneal microRNAs for the diagnosis of PD-related peritonitis.

microRNA	AUC	95% Confidence interval
miR-223	0.820	0.72–0.92
miR-27a	0.719	0.59–0.85
miR-21	0.726	0.59–0.86
miR-31	0.884	0.81–0.96
miR-223/miR-31 ratio	0.986	0.97–1.00

ROC curve analysis of the potential of individual microRNAs, as well as the ratio of miR-223/miR-31, to discriminate between stable and infected PD patients on the day of presentation with acute symptoms, based on relative microRNA levels. AUC, area under the curve.

### Peritoneal microRNA levels during the resolution of the infection

**Figure 2:**
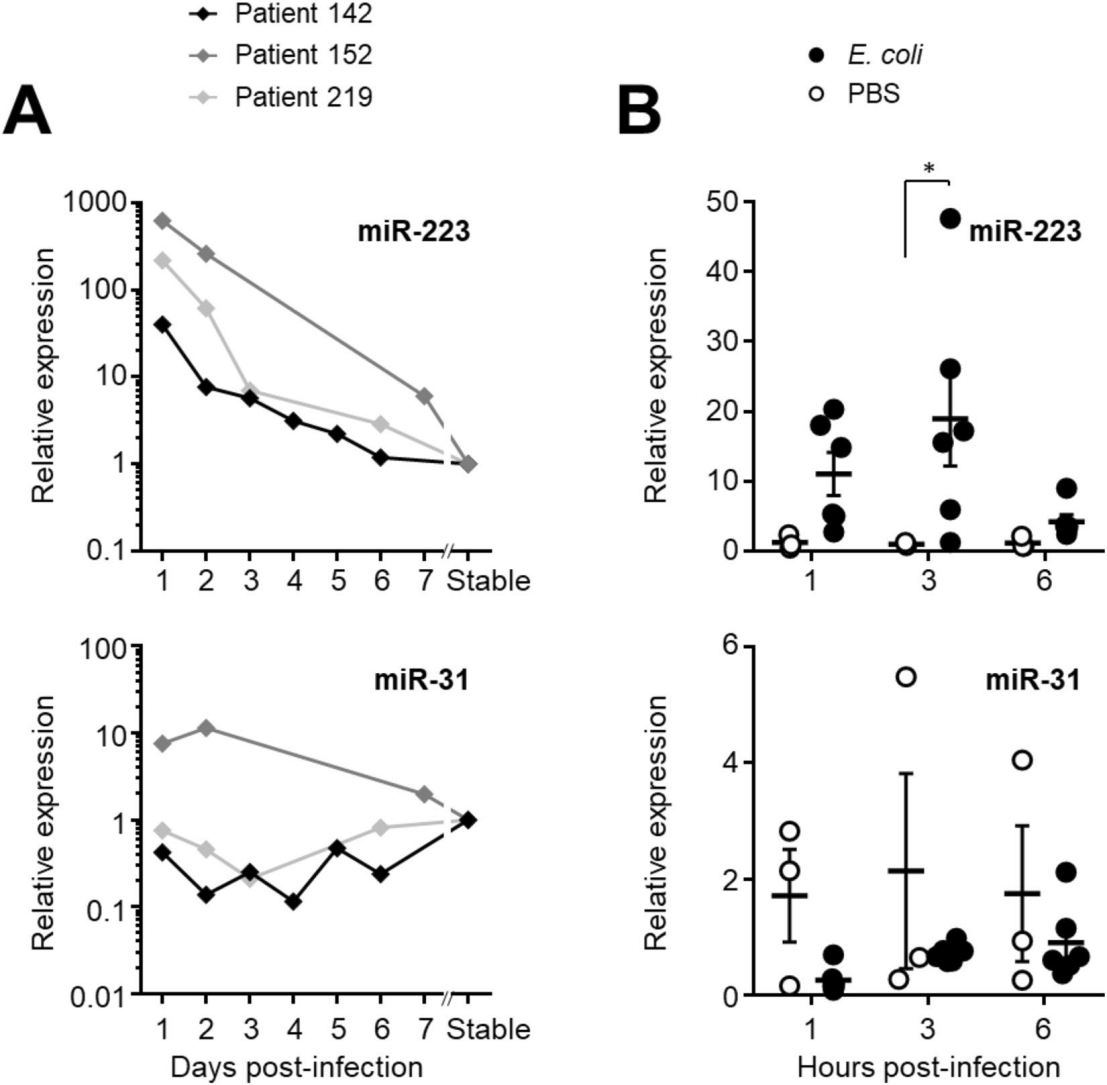
Peritoneal microRNAs during bacterial peritonitis. (**A**) miR-223, miR-21, miR-27a and miR-31 were measured in effluent samples from three individual patients followed over the course of a week, starting from their presentation at hospital with acute peritonitis. All levels were normalised to snRNA U6 levels and are shown as relative values compared to a corresponding post-infection sample from each patient 10–12 months after the resolution of the peritonitis episode. Infective organisms were identified by microbiological culture as *Staphylococcus aureus* (#142), coagulase-negative *Staphylococcus* (#152) and alpha-haemolytic *Streptococcus* (#219). (**B**) microRNA expression during acute peritoneal *E*. *coli* infection in C57BL/6 mice aged 8–12 weeks (*n* = 6), compared to mock-treated animals (PBS; *n* = 3). Each data point represents an individual animal; lines indicate means and standard errors. Data were analysed by two-way ANOVA and Holm-Sidak’s multiple comparisons test.

As the levels of miR-223 and miR-31 were markedly altered early in infection, the patterns of microRNA expression through the course of the peritonitis episode were investigated further. Using longitudinal collections of effluent samples from three PD patients over one week starting from the day of presentation with a cloudy effluent, both microRNAs were measured, normalised to snRNA U6, and compared to the pre-infection levels in stable samples from the same patients, before presenting with peritonitis. In line with the cross-sectional analysis shown in Fig. 1, miR-223 levels were strongly elevated on day 1 of the infection in all three patients, from where they declined rapidly to reach baseline levels by day 6–7 of the episode (Fig. 2A). miR-31 levels showed more variability across the three patients.

These findings were mirrored in an *in vivo* model that allowed us to study the time course of microRNA expression after the onset of infection, in the absence of other confounding factors^15^. Here, local levels of extracellular miR-223 were increased in infected mice as early as 1 hour after injection of *E*. *coli* and peaked at 3 hours, returning to near baseline within 6 hours (Fig. 2B). There was also a modest decrease in the levels of miR-31 in infected mice, similarly to infected patients. In summary, these findings suggested that miR-223 in particular shows similar kinetics in the response to peritoneal infections in humans and in mice and reaffirmed its potential as promising biomarker of bacterial infection. Moreover, as miR-223 levels decreased with the resolution of the immune reaction a future miR-223 test may also be relevant for monitoring the response to treatment.

### Cellular sources of extracellular microRNAs during peritonitis

**Figure 3:**
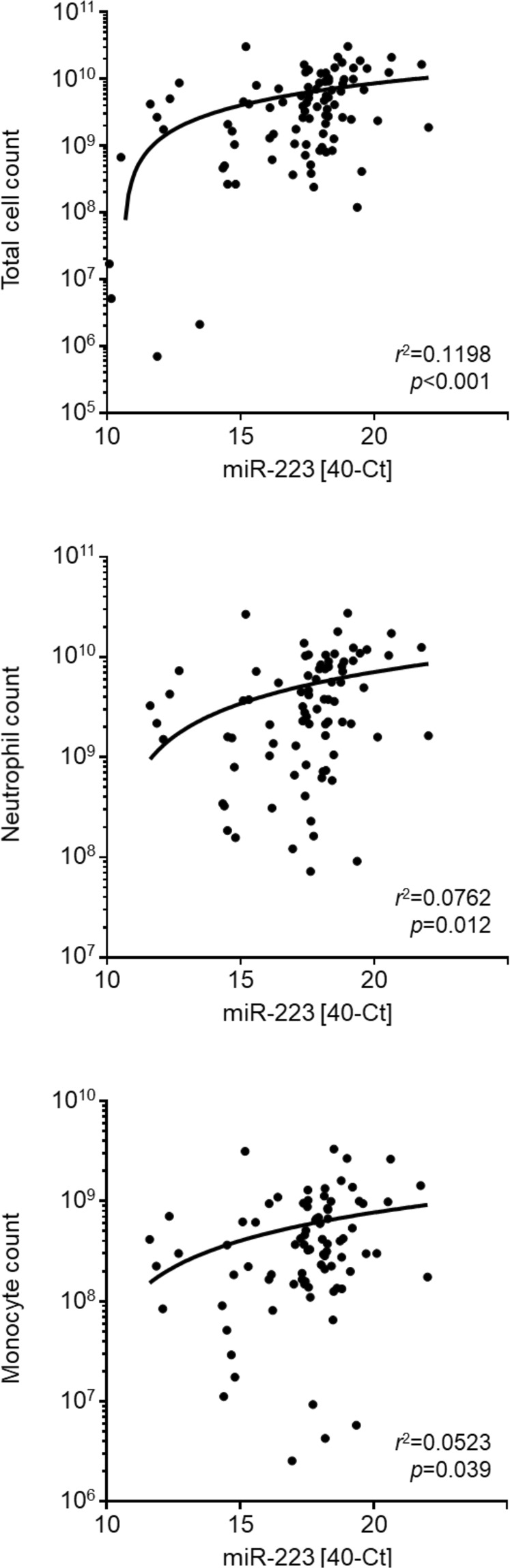
Correlation of peritoneal microRNAs with cellular infiltration during peritonitis. Linear regression of peritoneal miR-223 levels (40−Ct) with cell numbers in the inflammatory infiltrate, shown as total cell count and the number of live CD14^+^ monocytes/macrophages or CD15^+^ neutrophils in effluent samples from 82 PD patients with acute peritonitis.

We next sought to define the potential cellular source of extracellular microRNAs by identifying possible correlations of microRNA levels with the cellular infiltrate during acute peritonitis. To this end, data were analysed by linear regression for 82 patient samples, for which all necessary cellular and microRNA data were available. For all combinations, *r*^2^ values were very low, indicating that correlations were not strong. Nevertheless, significant correlations were found between miR-223 and numbers of total cells, macrophages and neutrophils (Fig. 3), suggesting that miR-223 may derive from the immune cells that infiltrate the peritoneum during infection. miR-27a showed a similar trend of correlations with the cellular levels but not miR-21 and miR-31 (data not shown).

**Figure 4:**
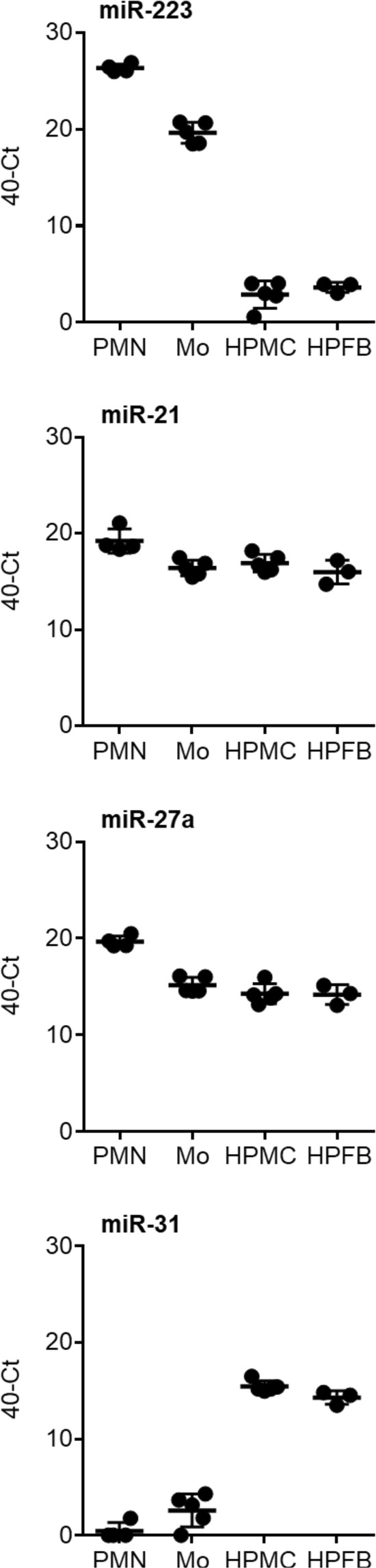
Cellular source of peritoneal microRNAs. Expression of microRNAs upon *in vitro* culture for 4 hours of neutrophils (PMN, *n* = 4) and monocytes (Mo, *n* = 5) from human blood and mesothelial cells (HPMC, *n* = 5) and fibroblasts (HPFB, *n* = 3) from human omentum. Each data point corresponds to an individual donor; lines indicate means and standard deviation.

In agreement with these observations, miR-223 was expressed at high levels by neutrophils and monocytes obtained from peripheral blood of healthy volunteers (Fig. 4). In contrast, expression of miR-223 by human peritoneal mesothelial cells (HPMCs) and human peritoneal fibroblasts (HPFBs) from omentum was low. miR-31 had the opposite expression pattern to miR-223. This microRNA was expressed at higher levels by HPMCs and HPFBs, while expression by neutrophils and monocytes was near or below the limit of detection (Fig. 4). In contrast, miR-27a and miR-21 were ubiquitously expressed at similar levels by all four cell types (data not shown). Taken together, these data demonstrated that miR-223 and miR-31 have cell-specific expression patterns, suggesting that during acute peritonitis miR-223 likely derived from infiltrating immune cells and miR-31 from local tissue cells.

### Identification of miR-223 in extracellular vesicles in PD effluent

**Figure 5:**
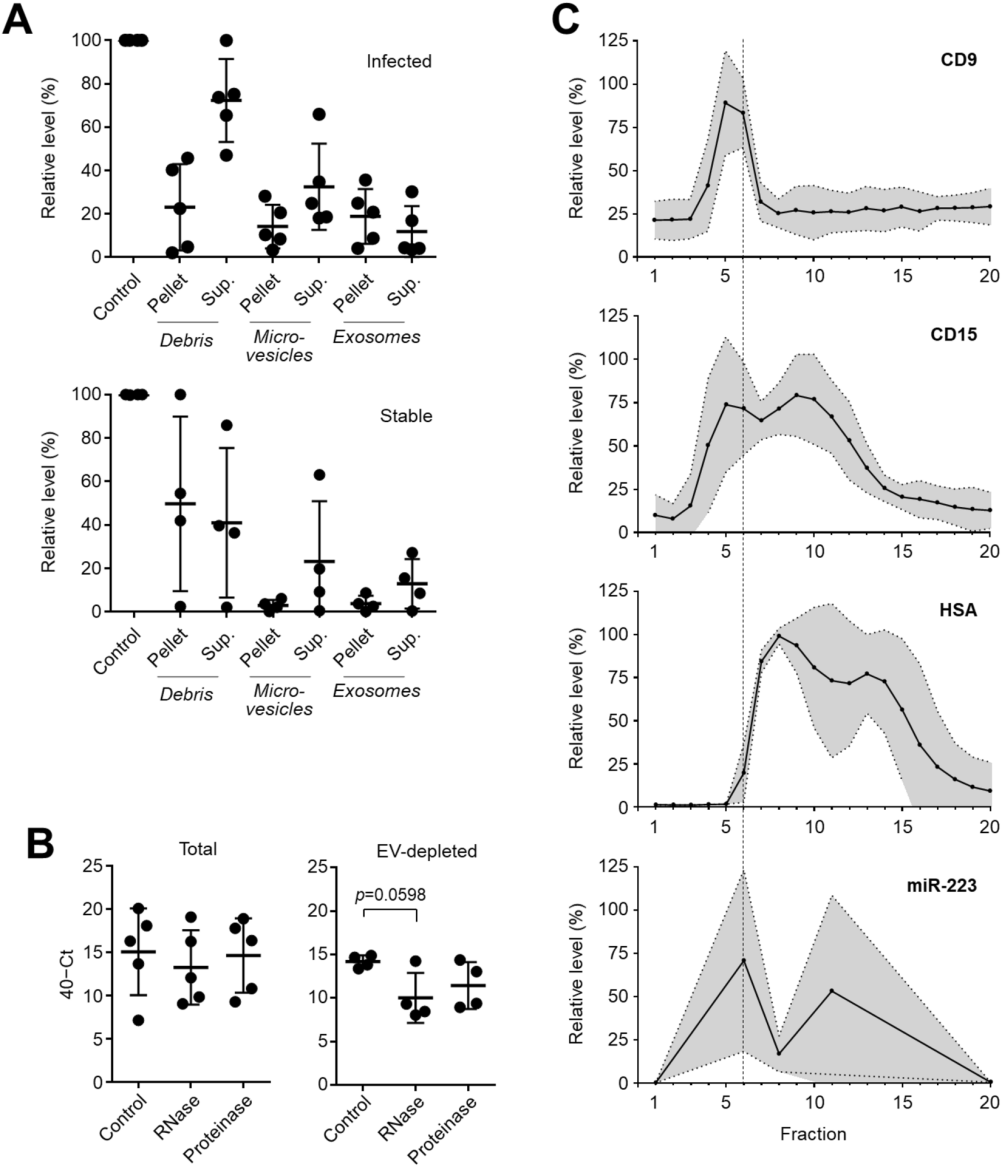
Stabilisation of miR-223 in PD effluent by extracellular vesicles. (**A**) miR-223 levels in cell-free effluent from infected (top, *n* = 5) and stable (bottom, *n* = 4) PD patients, before (control) and after differential centrifugation to pellet cellular debris, larger microvesicles and smaller exosomes. Data are shown as mean values ± SD, in relation to the amount of miR-223 present in the unspun effluent (control) serving as reference. (**B**) Susceptibility of extracellular miR-223 in effluent from infected PD patients to RNase A and proteinase K treatment, before (left, *n* = 5) and after (right, *n* = 4) depletion of exosomes. Each data point corresponds to an individual patient, shown as raw 40−Ct values; lines indicate means and standard deviations. Statistical analysis was performed using Kruskal-Wallis tests combined with Dunn’s multiple comparisons tests. (**C**) Fractionation of cell-free effluent from five infected PD patients by size exclusion chromatography and detection of CD9, CD15 and human serum albumin (HSA) in each fraction using plate-bound immunoassays. miR-223 was only quantified in fractions 1, 6 (marked by the dashed line), 11 and 20. Graphs depict the relative levels of each marker compared to the fraction containing the maximum amount; lines show the means and the shaded areas the 95% confidence interval.

To identify how extracellular miR-223 is stabilised in the peritoneal cavity during peritonitis, differential centrifugation was used to isolate fractions from cell-free effluent that were representative of cell debris, large extracellular vesicles (microvesicles) or small extracellular vesicles (exosomes). While miR-223 could be detected in all fractions there was a substantial amount of miR-223 present in the pellets from each centrifugation step, including the extracellular vesicle (EV) fraction (Fig. 5A). In contrast to the situation during acute peritonitis, miR-223 levels in the effluent of stable, non-infected PD patients were much lower, and barely detectable in pelleted fractions. In accordance with its presumed association with EVs, extracellular miR-223 in effluent from infected PD patients was resistant to both RNase A and proteinase K treatment (Fig. 5C), which is consistent with an intraluminal topology for the microRNA. Depletion of small extracellular vesicles by high speed centrifugation demonstrated that the residual extracellular miR-223 in the samples was partially susceptible to RNase degradation, suggesting that approx. 50% (ranging from <10% to >75%) of the extracellular miR-223 was associated with EVs, another proportion present as soluble unprotected molecule (Fig. 5C). Indeed, separation of effluent from infected PD patients by size exclusion chromatography demonstrated that a proportion of miR-223 co-segregated with the vesicle marker CD9 and with the neutrophil marker CD15, suggesting that at least some of the extracellular miR-223 might have been in neutrophil-derived exosomes (Fig. 5B). Probing for human serum albumin as control demonstrated that soluble proteins could clearly be separated from EVs, and that only a relatively small proportion of extracellular miR-223 co-segregated with free protein. Taken together, these experiments provided evidence that up to 75% of extracellular miR-223 co-elutes with EVs present in infected effluent including EVs that are positive for the neutrophil marker CD15.

## Discussion

Peritonitis is a serious complication of PD and a major cause of catheter removal, transition to an alternative renal replacement therapy, and mortality. The clinical problem is exacerbated by empirical, non-targeted treatments that cover the spectrum of likely organisms before culture results may become available, and by the current inability to undertake an accurate risk stratification and outcome prediction at the point of care that would guide early patient management^1–4^. The present single centre study provides evidence that suggests that local microRNAs might be useful as disease-specific biomarkers to help inform treatment decisions earlier and more accurately. The role of extracellular microRNAs as biomarkers has been explored in various biological fluids, including in PD effluent as correlates of peritoneal membrane fibrosis, but has so far not been applied to PD-related peritonitis. Here, miR-223 was identified as a promising biomarker associated with the inflammatory response during acute peritonitis that is characterised by a substantial infiltration of neutrophils into the peritoneal cavity. Other microRNAs of interest included miR-21, miR-27a and miR-31, with miR-21 being particularly relevant to distinguish between infections caused by Gram-positive and Gram-negative organisms.

Our data suggest that miR-223 is mainly released by local immune cells which infiltrate the peritoneum upon infection, corroborating earlier reports that this microRNA is predominantly expressed by cells from the myeloid lineage^16^. The high expression by infiltrating neutrophils and macrophages agrees with the large increase of miR-223 levels in cell-free PD effluent in acute peritonitis as well as in experimental peritonitis in mice, and the subsequent decay in parallel with the resolution of the inflammatory response. These findings evoke earlier studies implicating miR-223 as biomarker for diagnosis or progression in a range of conditions. For instance, miR-223 is increased in sepsis and rheumatoid arthritis, as well as in viral infections and animal models of bacterial infections^17,18^. Of relevance for this study, miR-223 in ascites fluid was reported previously to distinguish between spontaneous bacterial peritonitis and peritoneal carcinomatosis^19^, suggesting a discrimination between the inflammatory reactions to infection and other pathological scenarios. In this study, a large proportion of miR-223 in infected PD effluent was found to be stabilised by incorporation into EVs, as evidenced by ultracentrifugation, size-exclusion chromatography and resistance to RNase. Given the strong expression of miR-223 by cultured neutrophils and the abundance of neutrophils at the site of acute inflammation, together with the high levels of miR-223 in the peritoneal cavity of infected but not of stable PD patients, it is conceivable that these EVs may have been released by neutrophils in response to infection. Of note, EV-associated miR-223 was reported before as mediator in a range of inflammatory scenarios including macrophage differentiation and activation, cancer and lung epithelial cell inflammation^20–23^. It remains to be investigated whether the extracellular miR-223 in the PD effluent is functional and taken up by local immune and non-immune cells, or is merely a surrogate marker of the immune cell infiltration to the site of infection.

Another microRNA of interest, miR-31, was only expressed and released by resident peritoneal mesothelial cells and fibroblasts but not by neutrophils or monocytes. This restriction to tissue cells is in agreement with previous reports showing that miR-31 is predominantly an epithelial-specific microRNA^22^. miR-31 has been implicated in inflammatory pathways and conditions including psoriasis, inflammatory bowel disease, sepsis and allergy, with pleiotropic functions depending on the specific context and the local microenvironment. Amongst other roles, miR-31 regulates neutrophil recruitment in allergic airway disease and skews T cell responses^24–26^, and contributes to membrane fibrosis in PD^13^. The fact that miR-31 levels during acute peritonitis were lower than in the stable, non-infected peritoneum may point toward an inflammation-induced down-regulation of miR-31 expression in the peritoneal membrane. Alternatively, constitutively expressed miR-31 may simply be diluted out in early infection by the vast influx of immune cells and associated soluble mediators including microRNAs such as miR-223. A similar explanation may explain why levels of miR-21 and miR-27a are lower during peritonitis than in stable patients, two microRNAs that are expressed at similar levels by both resident tissues and infiltrating immune cells^27,28^.

Taken together, our findings suggest that exosome-encapsulated microRNAs may have a role in intercellular communication between immune cells responding to the infection and the local tissue, where they may help clear the infection, resolve the inflammation and restore homeostasis. While a further characterisation of this role was beyond the scope of the present study, we demonstrate that extracellular microRNAs are useful biomarkers of infection in PD-related peritonitis and have the potential to contribute to disease-specific immune fingerprints, subject to validation across multiple centres. Conventional detection of microRNAs typically requires lengthy protocols and specialised equipment, yet recent technological advances indicate that high levels of sensitivity can be achieved rapidly and cheaply using methods such as electrochemical detection^29^, and that the development of clinically and commercially viable point-of-care tests for microRNAs thus appears feasible. Such novel diagnostics are particularly attractive as early self tests in a patient’s home, at the onset of feeling unwell but before the full clinical manifestation of acute peritonitis^5,6^.

## Materials and Methods

### Study approval

All methods were carried out in accordance with relevant guidelines and regulations. Experimental protocols were approved by Cardiff University, and written informed consent was obtained from all subjects. Recruitment of PD patients and healthy volunteers for this study was approved by the South East Wales Local Ethics Committee under reference numbers 04WSE04/27 and 08/WSE04/17, respectively, and conducted according to the principles expressed in the Declaration of Helsinki. The PD study was registered on the UK Clinical Research Network Study Portfolio under reference numbers #11838 “Patient immune responses to infection in Peritoneal Dialysis” (PERIT-PD). Fresh omentum samples from consented patients were obtained from the Wales Kidney Research Tissue Bank.

Animal experimental protocols adhered to local and national guidelines. All animal studies were approved by the Animal and Welfare Ethical Review Body at Cardiff University and conducted inside the designated establishment at Cardiff University, which fully complies to the Home Office Code of Practice for the Housing and Care of Animals Bred, Supplied or Used for Scientific Purposes pursuant to the Animal (Scientific Procedures) Act, 1986.

### Peritoneal effluent

The study cohort comprised 107 adult PD patients admitted between September 2008 and October 2018 to the University Hospital of Wales, Cardiff, on day 1 of acute peritonitis, before commencing antibiotic treatment (34.6% female; median age 68 years, range 22–91 years). Three individual patients were followed over up to 7 days after presenting with peritonitis, and again 10–12 months after the resolution of the infection. 20 age and gender-matched individuals receiving PD and with no previous infections for at least 3 months served as stable, non-infected controls (35.0% female; median age 69.5 years, range 28–93 years). Subjects known to be positive for HIV or hepatitis C virus were excluded. Clinical diagnosis of acute peritonitis was based on the presence of abdominal pain and cloudy peritoneal effluent with >100 white blood cells/mm^3^. According to the microbiological analysis of the effluent by the routine Microbiology Laboratory, Public Health Wales, episodes of peritonitis were defined as culture-negative (with unclear aetiology) or as confirmed bacterial infections caused by Gram-positive and Gram-negative organisms. Cases of fungal infection and negative or unclear culture results were excluded from this analysis. Samples from ≥8 hour dwells with volumes of 1–2.5 liters were collected for biomarker measurements and processed as described before^8,9^.

### *In vivo* model of bacterial peritonitis

8–12-week-old C57BL/6 mice were injected intraperitoneally with 500 μl PBS or with live *E*. *coli* (ATCC 25923 strain, 2 × 10^7^ CFU per animal), as described in^15^. At the indicated time points, the mice were sacrificed and their peritoneal cavities were lavaged with 2 ml of ice-cold PBS. Lavage samples were rendered cell-free and subjected to RNA extraction.

### Cell isolation and culture

Human peritoneal mesothelial cells (HPMCs) and human peritoneal fibroblasts (HPFBs) were isolated from omental tissue of consented non-PD patients undergoing abdominal surgery, as described before^8,13^. MeT-5A, a human epithelial cell line, was purchased from the American Type Culture Collection (ATCC). Mesothelial cells and MeT-5A cells were cultured in Earle’s buffered Medium 199 (Life Technologies) containing 10% foetal calf serum, peritoneal fibroblasts were cultured in a 1:1 (*v*/*v*) mixture of DMEM and Ham’s F-12 nutrients (Life Technologies) with 20% foetal calf serum; both media were supplemented with 100 U/ml penicillin, 100 µg/ml streptomycin and 2 mM L-glutamine (Life Technologies) as well as 5 µg/ml apo-transferrin, 5 µg/ml bovine pancreas insulin and 0.4 µg/ml hydrocortisone (all from Sigma-Aldrich). Peripheral blood mononuclear cells (PBMCs) were isolated from venous blood of consented healthy volunteers using Ficoll-Paque (Axis-Shield). Monocytes (>95% CD14^+^) were purified from PBMC using anti-CD14 microbeads (Miltenyi). Neutrophils were isolated from venous blood of healthy volunteers after separation over Ficoll-Paque Plus (GE Healthcare). Erythrocyte contamination of the bottom layer was reduced by precipitation with 4% dextran and hypotonic lysis in 10 mM NaCl. High purity neutrophils (>99.5% CD15^+^ CD14^−^) were then isolated by negative selection using the EasySep Human Neutrophil Enrichment Kit (Stemcell Technologies)^30^. Monocytes and neutrophils were cultured in RPMI-1640 medium supplemented with 10% foetal calf serum, 50 µg/ml penicillin/streptomycin, 2 mM L-glutamine, 1% sodium pyruvate and 100 μM non-essential amino acids (all from Life Technologies).

### RNA extraction

RNA was extracted from cell-free liquid samples (PD effluent, culture supernatants, peritoneal lavages) using the mirVana PARIS and Native Protein Purification Kit (Life Technologies), using the manufacturer’s protocol to enrich for small RNAs. 100 pM of *Caenorhabditis elegans* miR-39 (cel-miR-39; Life Technologies) was spiked into each sample as internal reference. RNA from cells grown in standard tissue culture plates was extracted using TRIzol (Invitrogen) or RNeasy isolation kits (Qiagen). RNA yields were determined using a NanoDrop 2000 Spectrophotometer.

### TaqMan low density array

RNA was reverse transcribed to cDNA in MicroAmp 8-tube strips using the Megaplex RT Kit (Life Technologies), over 40 cycles of 2 min at 16 °C, 1 min at 42 °C and 1 sec at 50 °C, followed by 5 min at 85 °C and a cooling step at 4 °C. cDNA was then pre-amplified in MicroAmp 8-tube strips using the TaqMan PreAmp Kit (Life Technologies), for 10 min at 95 °C, 2 min at 55 °C and 2 min at 72 °C, followed by 12 cycles of 15 s at 95 °C and 4 min at 60 °C, an enzyme inactivation step of 10 min at 99.9 °C and a cooling step at 4 °C. Finally, pre-amplified cDNA was detected on a pre-loaded 384-well plate-based TaqMan Array Human MicroRNA A Card v2.0 (Life Technologies), using TaqMan Universal PCR Master Mix with No AmpErase UNG, and run on a ViiA 7 real-time PCR system (Life Technologies) with the 384 well TaqMan Low Density Array default thermal cycling conditions. Sample specific threshold cycle were calculated according to the 2^−∆∆Ct^ method. microRNA levels were normalised to the global levels across the plate, using standard settings.

### RT-qPCR

RNA was reverse transcribed to cDNA using the High-Capacity cDNA Reverse Transcription Kit (Life Technologies), MultiScribe Reverse Transcriptase (Life Technologies), RNase inhibitor (New England BioLabs), and microRNA specific primers (Life Technologies), for 10 min at 25 °C, 2 h at 37 °C and 5 s at 85 °C, followed by a cooling step at 4 °C. microRNA qPCR was carried out using TaqMan Universal PCR Master Mix, No AmpErase UNG (Life Technologies) with specific PCR primers (Life Technologies). Reactions were run on a ViiA7 real-time PCR System using Optical 96-Well Fast Plates (Life Technologies), for 10 min at 95 °C and then 40 cycles of 15 s at 95 °C and 1 min at 60 °C. Expression levels of microRNAs were normalised to the expression level of small nuclear RNA U6 (U6 snRNA) as internal control.

### Differential centrifugation for isolation of EVs

Samples were cleared of cells and cell debris with preliminary centrifugation steps at 300 *g* and 2,000 *g* respectively, each for 10 min at 4 °C. The supernatants from these steps were then centrifuged at 10,000 *g* for 1 h, at 4 °C to pellet larger EVs. Ultracentrifugation of the resulting supernatant at 100,000 *g* for 1 h, at 4 °C was used to pellet smaller EVs. Supernatants and pellets were each spiked with 100 pM of cel-miR-39 as internal reference, and treated as required with RNase A (Life Technologies) at 37 °C for 30 min, 50 μg/ml proteinase K (Sigma-Aldrich) at 55 °C for 30 min, or both, as described earlier^31^.

### Size exclusion chromatography

Pre-packed Exo-Spin size exclusion columns (Cell Guidance Systems) were washed with 6 mM EDTA in PBS, then 2 ml of PD effluent samples were added, and 15 × 1 ml aliquots were collected. RNA was extracted from 500 μl of each aliquot; the remainder was used for plate-based immuno-assays. Column fractions were bound overnight to protein-binding ELISA plates (Greiner Bio-One) and blocked with 1% (*w*/*v*) BSA in PBS. Bound material was detected with primary antibodies against CD9 (clone 209306; R&D Systems), CD15 (clone W6D3; BD Biosciences) or human serum albumin (clone 188835; R&D Systems), and developed using goat anti-mouse-biotinylated antibodies (Perkin Elmer) and europium-conjugated streptavidin (Perkin Elmer). Signals were measured by time-resolved fluorometry, using a Pherastar FS plate reader (BMG Labtech)^32^.

### Flow cytometry

Cells from freshly collected cloudy peritoneal effluents were acquired on an eight-colour FACSCanto II (BD Biosciences) and analyzed with FlowJo 10.1 (TreeStar), using monoclonal antibodies against CD3 (SK7) and CD15 (HI98 or HIM1) from BD Biosciences; and anti-CD14 (61D3) from eBioscience. Leukocyte populations were gated based on their appearance in side scatter and forward scatter area/height and exclusion of live/dead staining (fixable Aqua; Invitrogen).

### Statistics

Statistical analyses were performed using GraphPad Prism 6.0, as indicated in the figure legends. All variables were tested for normal distribution using D’Agostino-Pearson tests. All statistical tests were two-tailed; differences were considered significant as indicated in the figures and tables: *p < 0.05; **p < 0.01; ***p < 0.001.

## Supplementary information

Supplementary Information
