# Supplementary material for: Neutrophil-derived miR-223 as local biomarker of bacterial peritonitis
Source: Sci Rep. 2019 Jul 12;9:10136. doi: 10.1038/s41598-019-46585-y (PMC6625975; doi:10.1038/s41598-019-46585-y)
Supplement: Supplementary file 1 — Supplementary Information [file 41598_2019_46585_MOESM1_ESM.docx]

**Neutrophil-derived miR-223 as local biomarker of bacterial peritonitis**

**Amy C. Brook^1^, Robert H. Jenkins^1,2^, Aled Clayton^3^, Ann Kift-Morgan^1^,
Anne-Catherine Raby^1,2^, Alex P. Shephard^3^, Barbara Mariotti^4^, Simone M. Cuff^1^,
Flavia Bazzoni^4^, Timothy Bowen^1,2^, Donald J. Fraser^1,2,5,6^, Matthias Eberl^1,6,*^**

**Supplementary Information**

**Supplemental Table S1.** Patient characteristics of all individuals recruited to the present study. ID, patient number; PD, vintage time on peritoneal dialysis in years.

| **ID** | **Sex** | **PD**  **(yrs)** | **Age**  **(yrs)** | **Type** | **Microbiological culture result** |
| --- | --- | --- | --- | --- | --- |
| 088-02 | M | 3.76 | 79 | Stable | Never infected |
| 098-06 | M | 1.24 | 48 | Stable | Never infected |
| 113-01 | M | 1.06 | 68 | Stable | Never infected |
| 114-01 | M | 3.36 | 63 | Stable | Never infected |
| 128-06 | M | 1.00 | 71 | Stable | Never infected |
| 132-04 | M | 1.02 | 76 | Stable | Never infected |
| 132-15 | M | 3.21 | 77 | Stable | 9 months after last infection |
| 135-02 | F | 1.21 | 67 | Stable | Never infected |
| 140-01 | M | 1.58 | 62 | Stable | Never infected |
| 147-01 | M | 2.34 | 73 | Stable | Never infected |
| 171-02 | M | 1.60 | 65 | Stable | >2 years after last infection |
| 191-02 | M | 2.82 | 83 | Stable | Never infected |
| 201-07 | F | 1.37 | 49 | Stable | Never infected |
| 203-04 | M | 1.31 | 71 | Stable | Never infected |
| 215-02 | M | 2.30 | 62 | Stable | Never infected |
| 218-03 | F | 1.27 | 39 | Stable | Never infected |
| 219-02 | F | 1.29 | 73 | Stable | Never infected |
| 219-02 | F | 1.29 | 73 | Stable | Never infected |
| 221-02 | F | 1.08 | 75 | Stable | Never infected |
| 246-02 | F | 1.75 | 28 | Stable | 3 months after last infection |
| 181-21 | M | 3.74 | 74 | Gram-pos | Coagulase negative *Staphylococcus* |
| 114-07 | M | 8.36 | 68 | Gram-pos | Coagulase negative *Staphylococcus* |
| 012-02 | M | 4.56 | 65 | Gram-pos | Coagulase negative *Staphylococcus* |
| 080-02 | M | 5.07 | 51 | Gram-pos | Coagulase negative *Staphylococcus* |
| 184-02 | M | 0.53 | 80 | Gram-pos | Coagulase negative *Staphylococcus* |
| 188-04 | M | 0.97 | 73 | Gram-pos | Coagulase negative *Staphylococcus* |
| 136-01 | F | 5.01 | 54 | Gram-pos | Coagulase negative *Staphylococcus* |
| 009-01 | M | 1.66 | 54 | Gram-pos | Coagulase negative *Staphylococcus* |
| 025-01 | M | 0.96 | 70 | Gram-pos | Coagulase negative *Staphylococcus* |
| 035-01 | F | 0.57 | 36 | Gram-pos | Coagulase negative *Staphylococcus* |
| 045-01 | F | 7.76 | 59 | Gram-pos | Coagulase negative *Staphylococcus* |
| 152-04 | M | 2.26 | 78 | Gram-pos | Coagulase negative *Staphylococcus* |
| 184-03 | M | 0.67 | 80 | Gram-pos | Coagulase negative *Staphylococcus* |
| 181-14 | M | 2.94 | 76 | Gram-pos | Coagulase negative *Staphylococcus* |
| 244-02 | M | 0.52 | 75 | Gram-pos | Coagulase negative *Staphylococcus* |
| 107-01 | F | 3.73 | 62 | Gram-pos | Coagulase negative *Staphylococcus* |
| 143-02 | M | 4.62 | 83 | Gram-pos | Coagulase negative *Staphylococcus* |
| 152-08 | M | 3.59 | 79 | Gram-pos | Coagulase negative *Staphylococcus* |
| 154-01 | F | 3.97 | 67 | Gram-pos | Coagulase negative *Staphylococcus* |
| 179-07 | M | 1.20 | 69 | Gram-pos | Coagulase negative *Staphylococcus* |
| 215-04 | M | 2.92 | 63 | Gram-pos | Coagulase negative *Staphylococcus* |
| 167-01 | M | 0.22 | 44 | Gram-pos | Coagulase negative *Staphylococcus* |
| 126-02 | M | 1.08 | 33 | Gram-pos | Coagulase negative *Staphylococcus* |
| 150-01 | M | 0.83 | 83 | Gram-pos | Coagulase negative *Staphylococcus* |
| 184-01 | M | 0.37 | 79 | Gram-pos | Coagulase negative *Staphylococcus* |
| 181-01 | M | 1.07 | 74 | Gram-pos | Coagulase negative *Staphylococcus* |
| 256-01 | M | 0.19 | 70 | Gram-pos | Coagulase negative *Staphylococcus* |
| 244-01 | M | 0.44 | 74 | Gram-pos | Coagulase negative *Staphylococcus* |
| 098-17 | M | 3.89 | 51 | Gram-pos | Coagulase negative *Staphylococcus* |
| 197-01 | F | 0.12 | 60 | Gram-pos | Coagulase negative *Staphylococcus* |
| 188-08 | M | 1.88 | 74 | Gram-pos | Coagulase negative *Staphylococcus* |
| 154-02 | F | 4.33 | 67 | Gram-pos | Coagulase negative *Staphylococcus* |
| 154-04 | F | 5.54 | 68 | Gram-pos | Coagulase negative *Staphylococcus* |
| 082-04 | F | 8.14 | 50 | Gram-pos | Coagulase negative *Staphylococcus* |
| 115-03 | M | 3.80 | 91 | Gram-pos | Coagulase negative *Staphylococcus* |
| 080-01 | M | 4.22 | 50 | Gram-pos | Coagulase negative *Staphylococcus* |
| 068-04 | M | 1.67 | 71 | Gram-pos | Coagulase negative *Staphylococcus* |
| 077-01 | F | 5.88 | 72 | Gram-pos | Coagulase negative *Staphylococcus* |
| 002-01 | M | 12.54 | 64 | Gram-pos | Coagulase negative *Staphylococcus epidermidis* |
| 188-05 | M | 1.06 | 73 | Gram-pos | Coagulase negative *Staphylococcus epidermidis* |
| 039-02 | M | 4.83 | 84 | Gram-pos | *Staphylococcus aureus* |
| 070-01 | M | 0.87 | 79 | Gram-pos | *Staphylococcus aureus* |
| 068-08 | M | 3.08 | 73 | Gram-pos | *Staphylococcus aureus* |
| 098-16 | M | 3.89 | 51 | Gram-pos | *Staphylococcus aureus* |
| 188-07 | M | 1.39 | 73 | Gram-pos | *Staphylococcus aureus* |
| 098-25 | M | 4.93 | 52 | Gram-pos | *Staphylococcus aureus* |
| 061-02 | M | 6.02 | 77 | Gram-pos | *Staphylococcus aureus* |
| 098-12 | M | 2.80 | 50 | Gram-pos | *Staphylococcus aureus* |
| 056-03 | F | 0.32 | 39 | Gram-pos | *Staphylococcus aureus* (MRSA) |
| 027-01 | F | 0.58 | 69 | Gram-pos | *Corynebacterium amycolatum* |
| 037-01 | F | 5.07 | 47 | Gram-pos | Coryneform bacteria |
| 040-02 | F | 4.74 | 62 | Gram-pos | *Enterococcus faecalis* |
| 150-02 | M | 0.91 | 83 | Gram-pos | Vancomycin resistant *Enterococcus* (VRE) |
| 150-03 | M | 0.97 | 83 | Gram-pos | Vancomycin resistant *Enterococcus* + *Enterococcus faecium* |
| 023-01 | M | 2.52 | 84 | Gram-pos | Microaerophilic *Streptococcus* |
| 018-02 | M | 1.06 | 60 | Gram-pos | Microaerophilic *Streptococcus* |
| 004-01 | M | 4.60 | 70 | Gram-pos | Alpha haemolytic *Streptococcus* |
| 019-02 | M | 2.83 | 64 | Gram-pos | Alpha haemolytic *Streptococcus* |
| 039-01 | M | 4.64 | 84 | Gram-pos | Alpha haemolytic *Streptococcus* |
| 070-10 | M | 1.95 | 80 | Gram-pos | Alpha haemolytic *Streptococcus* |
| 116-06 | M | 9.15 | 68 | Gram-pos | Alpha haemolytic *Streptococcus* |
| 156-01 | M | 1.22 | 73 | Gram-pos | Alpha haemolytic *Streptococcus* |
| 219-03 | F | 1.63 | 73 | Gram-pos | Alpha haemolytic *Streptococcus* |
| 023-03 | M | 4.89 | 86 | Gram-pos | Alpha haemolytic *Streptococcus* |
| 019-01 | M | 1.49 | 63 | Gram-pos | Alpha haemolytic *Streptococcus* |
| 294-01 | F | 0.80 | 42 | Gram-pos | Alpha haemolytic *Streptococcus* |
| 135-04 | F | 1.62 | 68 | Gram-pos | *Streptococcus sanguinis* |
| 237-01 | F | 0.24 | 33 | Gram-pos | *Streptococcus sanguinis* |
| 227-01 | F | 0.38 | 59 | Gram-pos | *Streptococcus B* |
| 013-01 | M | 2.12 | 75 | Gram-pos | Alpha haemolytic *Streptococcus*, non-haemolytic + coliform streptococci |
| 179-06 | M | 1.13 | 69 | Gram-pos | Coagulase neg *Staphylococcus*, Alpha haemolytic *Streptococcus* & *Propionibacterium* |
| 021-01 | M | 1.75 | 66 | Gram-pos | Gram-positive bacillus & *Corynebacterium*/*Diphtheroids* |
| 012-01 | M | 1.67 | 62 | Gram-pos | Coagulase negative *Staphylococcus* & *Corynebacterium* |
| 040-01 | F | 4.61 | 62 | Gram-pos | Coagulase negative *Staphylococcus* & *Enterococcus* |
| 159-01 | M | 0.06 | 44 | Gram-pos | *Enterococcus faecalis* + *Streptococcus bovis* |
| 181-07 | M | 2.23 | 75 | Gram-pos | *Corynebacterium jeikeium* |
| 016-01 | M | 0.79 | 76 | Gram-neg | *Acinetobacter* |
| 189-01 | F | 0.56 | 50 | Gram-neg | *Acinetobacter baumannii* |
| 238-09 | F | 1.81 | 59 | Gram-neg | *Acinetobacter ursingii* |
| 175-01 | M | 0.94 | 80 | Gram-neg | *Enterobacter* |
| 196-01 | M | 0.18 | 67 | Gram-neg | *Enterobacter* |
| 147-07 | M | 3.66 | 74 | Gram-neg | *Escherichia coli* |
| 136-04 | F | 5.30 | 55 | Gram-neg | *Escherichia coli* |
| 132-09 | M | 2.45 | 77 | Gram-neg | *Escherichia coli* |
| 143-03 | M | 4.99 | 84 | Gram-neg | *Escherichia coli* |
| 156-03 | M | 1.60 | 74 | Gram-neg | *Escherichia coli* |
| 180-02 | F | 1.10 | 64 | Gram-neg | *Escherichia coli* |
| 020-01 | F | 0.33 | 51 | Gram-neg | *Escherichia coli* |
| 031-01 | F | 3.34 | 64 | Gram-neg | *Escherichia coli* |
| 136-03 | F | 5.25 | 55 | Gram-neg | *Escherichia coli* |
| 117-06 | F | 0.66 | 36 | Gram-neg | *Escherichia coli* |
| 164-02 | F | 10.24 | 73 | Gram-neg | *Escherichia coli* |
| 238-02 | F | 0.80 | 58 | Gram-neg | *Escherichia coli* |
| 136-02 | F | 5.20 | 55 | Gram-neg | *Escherichia coli* |
| 266-01 | F | 0.24 | 22 | Gram-neg | *Escherichia coli* |
| 015-01 | M | 0.65 | 56 | Gram-neg | *Escherichia coli* |
| 210-12 | M | 3.14 | 85 | Gram-neg | *Escherichia coli* |
| 250-01 | F | 0.50 | 62 | Gram-neg | *Escherichia coli* + Gram-negative bacillus |
| 179-08 | M | 1.26 | 69 | Gram-neg | *Klebsiella* |
| 227-05 | F | 0.81 | 59 | Gram-neg | *Neisseria* |
| 054-03 | F | 0.19 | 82 | Gram-neg | *Morganella morganii* |
| 022-01 | M | 1.80 | 73 | Gram-neg | *Pseudomonas aeruginosa* |
| 029-01 | M | 7.38 | 75 | Gram-neg | *Pseudomonas aeruginosa* |
| 022-02 | M | 1.80 | 73 | Gram-neg | *Pseudomonas aeruginosa* |
| 084-01 | M | 1.01 | 90 | Gram-neg | Gram-negative bacillus |
| 248-01 | F | 1.04 | 79 | Gram-neg | *Acinetobacter* + Gram-positive bacillus |
| 174-01 | F | 0.20 | 72 | Gram-neg | *Pseudomonas aeruginosa* + Anaerobic Gram-negative bacillus |
| 142-04 | M | 4.87 | 64 | Gram-pos | *Staphylococcus aureus* |
| 142-08 | M | 5.89 | 65 | Stable | 12 months after last infection |
| 152-08 | M | 3.59 | 79 | Gram-pos | Coagulase negative *Staphylococcus* |
| 152-13 | M | 4.31 | 80 | Stable | 12 months after last infection |
| 219-03 | F | 1.63 | 73 | Gram-pos | Alpha haemolytic *Streptococcus* |
| 219-08 | F | 2.32 | 73 | Stable | 10 months after last infection |

**Supplemental Figure S1.** **Ratio of peritoneal miR-223 / miR-31 levels in PD patients.** (***A***) Ratios in 20 stable PD patients and 109 patients with confirmed bacterial infections. Data were analysed using a Mann-Whitney U test. (***B***) ROC curve analysis of the potential of the miR-223 / miR-31 to discriminate between stable and infected patients. AUC, area under the curve; CI, confidence interval.

**Supplemental Figure S2.** **Peritoneal miR-21 levels in PD patients presenting with a cloudy effluent.** (***A***) Levels of miR-21 in 76 patients with confirmed Gram^+^ infections and 31 with Gram^−^ infections, normalised to snRNA U6. Data were analysed using a Mann-Whitney U test. (***B***) ROC curve analysis of the potential of miR-21 to discriminate between Gram^+^ and Gram^−^ infections. AUC, area under the curve; CI, confidence interval.
